# Comparison of the efficacy and safety of baloxavir versus those of oseltamivir in pediatric patients with influenza: a meta-analysis

**DOI:** 10.3389/fmicb.2025.1672925

**Published:** 2025-10-28

**Authors:** Sheng Chen, Lingling Hua

**Affiliations:** ^1^Department of Pediatrics, The Affiliated Lihuili Hospital of Ningbo University, Ningbo, China; ^2^Department of Pediatrics, Women and Children’s Hospital of Ningbo University, Ningbo, China

**Keywords:** baloxavir, oseltamivir, influenza, efficacy and safety, meta-analysis

## Abstract

**Objective:**

Baloxavir is a ribonucleic acid polymerase inhibitor that effectively alleviates influenza symptoms in adults and adolescents. This meta-analysis aimed to comprehensively compare the efficacy and safety of baloxavir with those of oseltamivir in pediatric patients with influenza.

**Methods:**

A comprehensive study search was performed by encompassing multiple electronic databases, including PubMed, Web of Science, Embase, the Cochrane Library, Wan Fang, VIP, and CNKI, from database establishment to June 2025. Studies comparing the efficacy and safety of baloxavir and oseltamivir in pediatric patients with influenza were included.

**Results:**

A total of 10 studies, including 2,106 patients receiving baloxavir and 2,567 patients receiving oseltamivir, were included in the analysis. The pooled analysis indicated that baloxavir resulted in a shorter duration of symptoms and duration of fever than did oseltamivir (both *p* < 0.01). In the subgroup analysis, the statistical significance of the duration of symptoms remained unchanged in the subgroups of influenza types A and B, as well as in influenza A subtypes H1N1pdm09 and H3N2A (all *p* < 0.05). However, in terms of the duration of fever, the advantage of baloxavir over oseltamivir was observed in the influenza type A (*p* < 0.001) and B (*p* < 0.001) subgroups and in the influenza A subtype H1N1pdm09 (*p* < 0.001) subgroup but not in the H3N2A subgroup (*p* = 0.430). The incidence of any AE was similar between the two groups (*p* = 0.260). The quality of the enrolled studies was high, and there was no publication bias.

**Conclusion:**

In pediatric patients with influenza, baloxavir results in a shorter duration of symptoms and fever compared to oseltamivir, which may be attributed to their different bioavailability and half-lives. Additionally, the safety profiles of baloxavir and oseltamivir are comparable.

**Systematic review registration:**

https://www.crd.york.ac.uk/, CRD420251128843.

## 1 Introduction

Influenza, which is complicated by multisystem symptoms such as nasal discharge, fever, cough, and headache, commonly occurs in pediatric patients (Wolf and Antoon, 2023; Vasilakopoulos and Kainth, 2025; Goldman, 2021). In China, the incidence of influenza is estimated to range from 233.3 to 3744.79 per 1,000,000 people, varying across different regions (Liu et al., 2022; Wu et al., 2023). Even if influenza is cured spontaneously, a poor prognosis might occur if pediatric patients are complicated with diseases involving other organs, such as pneumonia, myocarditis, and pericarditis (Hao et al., 2025; Lee et al., 2024). The mortality rate reached 27.66 per 100,000 person-years in China from 2010 to 2015 (Jin et al., 2020). Currently, the main treatment modalities for influenza include neuraminidase inhibitors (such as oseltamivir, zanamivir, and peramivir). Among these, the degree of viral resistance to neuraminidase inhibitors is low, while these drugs have a relatively low genetic barrier to antiviral resistance (Zhang et al., 2025; Gao et al., 2025; Sato, 2025).

Baloxavir, a ribonucleic acid (RNA) polymerase inhibitor, has an anti-influenza effect by blocking the synthesis of viral mRNA (Dufrasne, 2021; Kuo et al., 2021; Shirley, 2020). A previous Phase 2 study indicated that baloxavir achieves a shorter time to alleviation of influenza symptoms than the placebo, indicating its superior efficacy in treating influenza in adults and adolescents (Hayden et al., 2018). However, it remains challenging to draw a definitive conclusion regarding the efficacy and safety of baloxavir in pediatric patients due to the inconsistent results (Ge et al., 2024; Baker et al., 2020). For example, one study reported that the mean duration of fever was shorter in the baloxavir group than in the oseltamivir group (Ge et al., 2024). However, another study revealed that the median time to alleviation of signs and symptoms of influenza was similar between the baloxavir and oseltamivir groups (Baker et al., 2020). Therefore, performing a meta-analysis to comprehensively compare the efficacy and safety of baloxavir and oseltamivir to provide fundamental evidence for the application of baloxavir in treating pediatric patients with influenza is essential.

Hence, we searched for studies in multiple electronic databases, including PubMed, Web of Science, Embase, the Cochrane Library, Wan Fang, VIP, and CNKI, to compare the efficacy and safety of baloxavir and oseltamivir in pediatric patients with influenza.

## 2 Methods

### 2.1 Search

A comprehensive study search was performed following the PRISMA (Preferred Reporting Items for Systematic Reviews and Meta-Analyses) guidelines to screen relevant studies comparing the efficacy and safety of baloxavir marboxil (baloxavir) versus oseltamivir in pediatric influenza patients. The search encompassed multiple electronic databases, including PubMed, Web of Science, Embase, the Cochrane Library, Wan Fang, VIP, and CNKI, from database establishment to June 2025. The search strategy employed a combination of free-text keywords, such as “baloxavir marboxil” OR “baloxavir” OR “Xofluza” OR “S-033188” OR “BXM,” “child*” OR “pediatri*,” and “influenza” OR “flu.” For example, the PubMed search was conducted as follows: (((Baloxavir marboxil OR Xofluza OR S-033188 OR BXM)) AND ((Child* OR Pediatri* OR Adolescent))) AND (Influenza OR Flu)); the Web of Science search used the following strategy: ((TS = (Baloxavir marboxil OR Xofluza OR S-033188 OR BXM)) AND TS = (Child* OR Pediatri* OR Adolescent) AND TS = (Influenza OR Flu). Similar strategies were applied to the other databases. Additionally, a manual search of reference lists from relevant reviews and meta-analyses was performed to ensure that no eligible studies were overlooked. This study was registered on PROSPERO (available at https://www.crd.york.ac.uk/) with the approval number CRD420251128843.

### 2.2 Inclusion and exclusion criteria

On the basis of the PICOS principle, studies were included if they met the following criteria: (1) population (P): pediatric patients with confirmed influenza infection; (2) intervention (I): treatment with baloxavir; (3) comparator (C): treatment with oseltamivir; (4) outcomes (O): duration of symptoms (defined as the time from treatment to influenza symptom resolution, e.g., cough, nasal congestion, sore throat, etc.), duration of fever (defined as the time from treatment to fever resolution), and incidence of any adverse events (AEs); and (5) study design (S): no restrictions were imposed on study design. The exclusion criteria were as follows: (1) studies involving adults only or without pediatric population analysis; (2) case reports, experimental studies, or systematic reviews; (3) studies without data for meta-analysis; (4) overlapping populations; and (5) studies not published in English or Chinese.

### 2.3 Data preparation and quality assessment

Data preparation was performed through a standardized form, extracting the following: (1) study information (first author, publication year, region, study design, and sample size); (2) patient information (age, sex, vaccination, influenza type/subtype, time from symptom onset to drug administration, and duration of medication use); and (3) outcome measures (duration of symptoms, duration of fever, and any AE). For the outcome measures, Saito et al. (2020), Sun et al. (2024), and Wagatsuma et al. (2022) did not report overall viral infection but reported several individual viral infection outcomes, and those individual viral infection outcomes were included separately in the primary analyses. Two investigators were responsible for the acquisition, analysis, and interpretation of the data. If a contradictory opinion was expressed, a discussion was held to reach a final conclusion.

The Cochrane risk of bias tool (2.0) was used to evaluate randomized controlled trials (RCTs) involving randomization, intervention, missing data, outcome, and reported bias (every domain assessed as low, high, or unclear risk) (Sterne et al., 2019). The Newcastle–Ottawa scale was used to evaluate non-RCTs, covering aspects such as selection, comparability, and outcome assessment (the maximum number of stars was 9, and a score greater than six stars was considered high quality) (Shi et al., 2020).

### 2.4 Statistical analysis

The meta-analysis was conducted via R version 4.3.3. For the “duration of symptoms” and “duration of fever,” standard mean differences (SMDs) with 95% confidence intervals (CIs) were calculated. For “any AE,” the risk ratio (RR) with 95% CI was used to pool statistics. Heterogeneity was assessed via *I*^2^ statistics (*I*^2^ > 50% indicating substantial heterogeneity). A random effect model was applied if heterogeneity was present; otherwise, a fixed effect model was used. Subgroup analyses were conducted on the basis of influenza virus type (A vs. B) and influenza virus A subtype (H1N1pdm09 vs. H3N2). Publication bias was evaluated via funnel plots and Begg’s test. Sensitivity analyses were performed by excluding studies one by one to assess robustness. A *p*-value <0.05 indicated significance.

## 3 Results

### 3.1 Study flow

**Figure 1:**
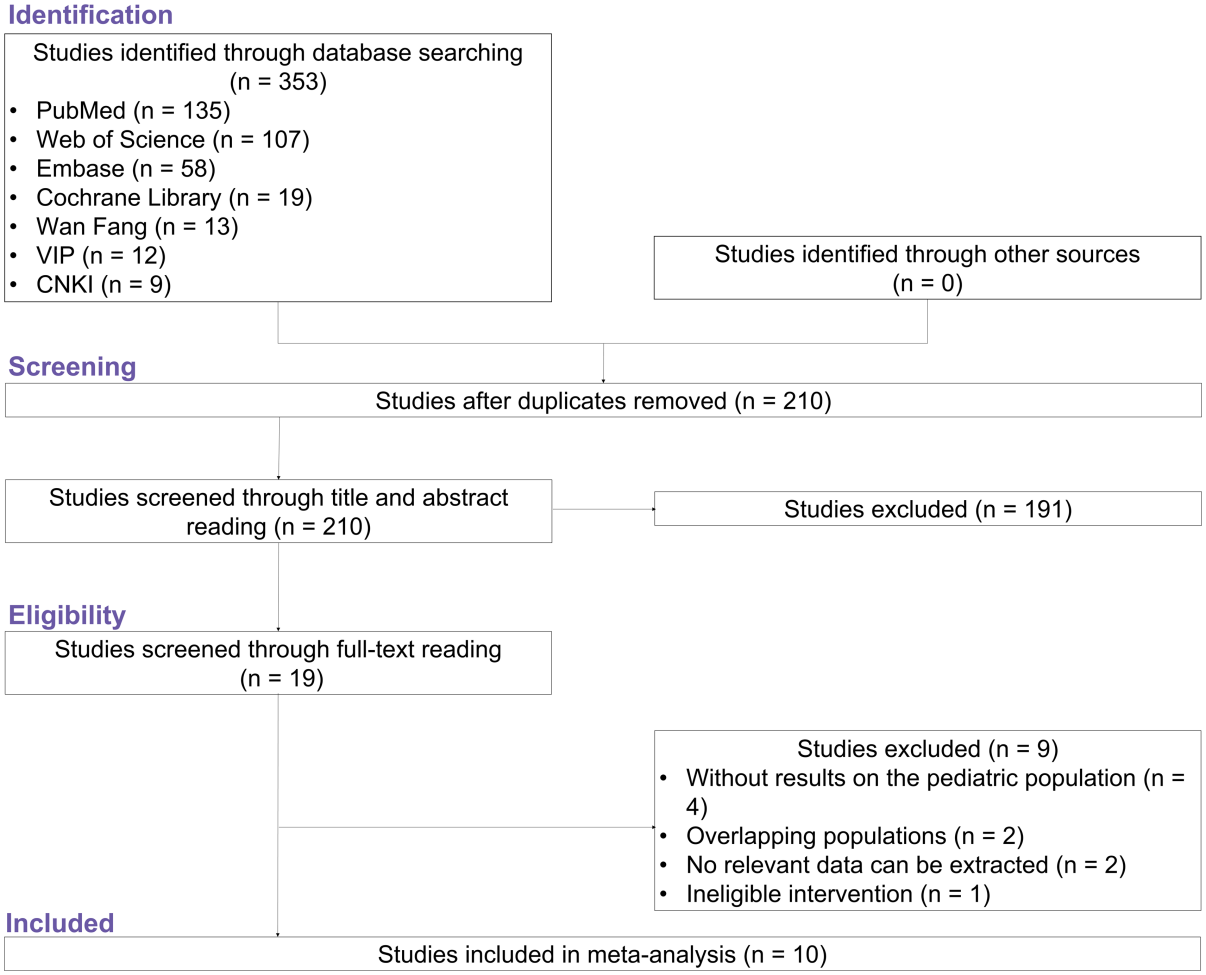
Study flow.

After identifying studies from multiple databases, a total of 353 studies were found, whereas no studies from other sources were identified. A total of 210 studies were subsequently screened by reading the title and abstract after removing duplicate papers. A total of 191 papers were excluded because they were not related to the topic, and 19 papers met the eligibility criteria. Among them, nine papers were excluded because they did not include a pediatric population (*n* = 4), had an overlapping population (*n* = 2), lacked relevant extractable data (*n* = 2), or were ineligible for intervention (*n* = 1). Finally, 10 studies (Ge et al., 2024; Baker et al., 2020; Saito et al., 2020; Sun et al., 2024; Wagatsuma et al., 2022; Chong et al., 2021; Ishiguro et al., 2025; Kakuya et al., 2022; Nezu et al., 2023; Su et al., 2024) were included in the analysis (Figure 1).

### 3.2 Information on the included studies

Among these 10 included studies, the publication dates ranged from 2020 to 2024. Seven studies were conducted in Japan, two studies were conducted in China, and one study was conducted in multiple countries. Eight studies were non-RCT studies, while two studies were RCT studies. A total of 2,106 patients received baloxavir, while 2,567 patients received oseltamivir. Other information, including sex, vaccination status, and influenza virus type, is presented in Table 1. Furthermore, the age, time from symptom onset to drug administration, and duration of medication use are shown in Supplementary Table 1.

**Table 1:** Information on the included studies.

No. of study	First author	Publication year	Region	Study design	Sample size (cases)		Sex (male/female, cases)		Vaccination (cases)		Influenza virus type
					Baloxavir	Oseltamivir	Baloxavir	Oseltamivir	Baloxavir	Oseltamivir	
S1 (Baker et al., 2020)	Baker J.	2020	USA, Poland, Spain, Costa Rica, Mexico, and Russia	RCT	115	58	55/60	26/32	59	26	A, B, mixed, and unknown
S2 (Chong et al., 2021)	Chong Y.	2021	Japan	Non-RCT	26	26	NA	NA	NA	NA	A
S3 (Ge et al., 2024)	Ge X.	2024	China	Non-RCT	420	445	256/164	243/202	NA	NA	A
S4 (Ishiguro et al., 2025)	Ishiguro N.	2025	Japan	RCT	128	67	66/62	38/29	26	18	A, B, mixed, and unknown
S5 (Kakuya et al., 2022)	Kakuya F.	2022	Japan	Non-RCT	144	91	83/61	43/48	80	43	A and B
S6 (Nezu et al., 2023)	Nezu K.	2023	Japan	Non-RCT	555	556	276/279	281/275	227	359	A and B
S7 (Saito et al., 2020)	Saito R.	2020	Japan	Non-RCT	102	52	56/46	23/29	15	7	A
S8 (Sun et al., 2024)	Sun Y.	2024	Japan	Non-RCT	150	926	84/66	499/427	NA	NA	A and B
S9 (Wagatsuma et al., 2022)	Wagatsuma K.	2022	Japan	Non-RCT	100	59	53/47	35/24	34	25	A and B
S10 (Su et al., 2024)	Su Z.	2024	China	Non-RCT	120	124	69/51	65/59	20	25	A and unknown

RCT, randomized controlled trial; NA, not available.

### 3.3 Comparison of the efficacy of baloxavir and oseltamivir

**Figure 2:**
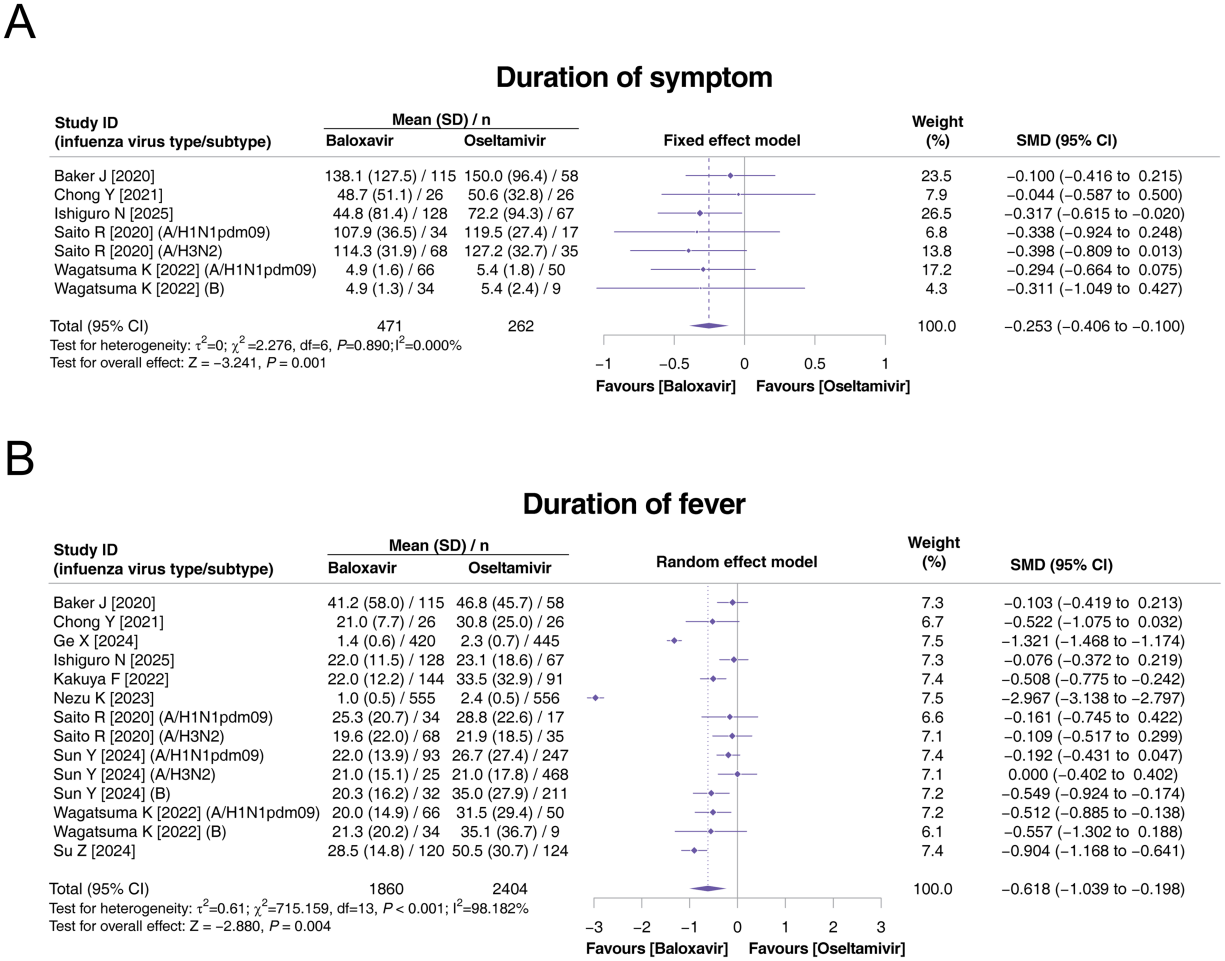
Comparison of the duration of symptoms and duration of fever between baloxavir and oseltamivir in pediatric patients with influenza. Pooled analysis of the duration of symptoms **(A)** and duration of fever **(B)** between baloxavir and oseltamivir in pediatric patients with influenza.

In terms of the duration of symptoms, seven studies assessed this outcome, and there was no heterogeneity among these studies (*p* = 0.890, *I*^2^ = 0.000%). The duration of symptoms was estimated to reach 73.91 ± 93.36 h in the baloxavir group and 82.65 ± 86.08 h in the oseltamivir group. Compared with oseltamivir, baloxavir was associated with a shorter duration of symptoms (SMD: −0.253, 95% CI: −0.406 to −0.100, *p* = 0.001; Figure 2A). With respect to the duration of fever, all 10 studies reported this endpoint, and there was heterogeneity among these studies (*p* < 0.001, *I*^2^ = 98.182%). The duration of fever was estimated to be 12.77 ± 20.77 h in the baloxavir group and 17.44 ± 23.83 h in the oseltamivir group. Compared with oseltamivir, baloxavir was associated with a shorter duration of fever (SMD: −0.618, 95% CI: −1.039 to −0.198, *p* = 0.004; Figure 2B).

### 3.4 Sensitivity analysis by subgroup analysis based on influenza virus type

Sensitivity analyses of the duration of symptoms and duration of fever by subgroup analysis based on influenza virus type (A or B) and influenza virus A subtype (H1N1pdm09 or H3N2) were carried out.

**Figure 3:**
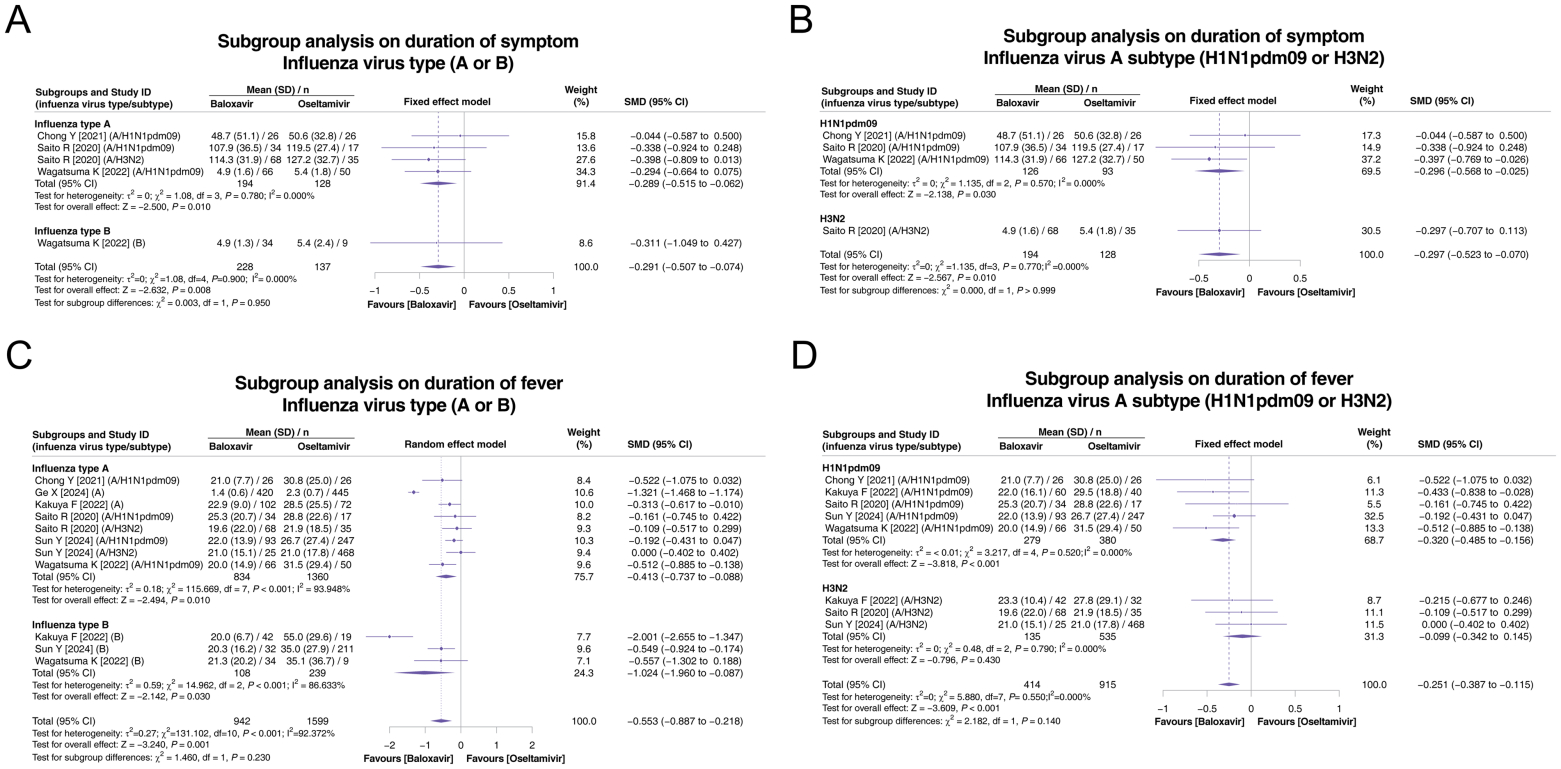
Subgroup analysis of the duration of symptoms and duration of fever according to influenza virus type (A or B) and influenza virus type A subtype (H1N1pdm09 or H3N2). Pooled analysis of symptom duration based on influenza virus type (A or B) **(A)** and influenza virus type A subtype (H1N1pdm09 or H3N2) **(B)**. Pooled analysis of the duration of fever based on influenza virus type (A or B) **(C)** and influenza virus type A subtype (H1N1pdm09 or H3N2) **(D)**.

Among the subgroups of influenza types A and B, 3 and 1 studies, respectively, reported the duration of symptoms. There was no heterogeneity in these two subgroups (both *p* > 0.05). The duration of symptoms was shorter in the baloxavir group than in the oseltamivir group in both subgroups (both *p* < 0.05), and there was no subgroup difference between these two subgroups (*p* = 0.950, Figure 3A). Among the subgroups of influenza virus A subtypes H1N1pdm09 and H3N2A, 3 and 1 studies, respectively, reported the duration of symptoms. There was no heterogeneity in these two subgroups (both *p* > 0.05). This outcome was shorter in the baloxavir group than in the oseltamivir group in both subgroups (both *p* < 0.05), and there was no subgroup difference between these two subgroups (*p* > 0.999, Figure 3B).

In the influenza type A and B subgroup analyses on the duration of fever, 6 and 3 studies, respectively, reported this endpoint. There was heterogeneity in these two subgroups (both *p* < 0.001). The duration of fever was shorter in the baloxavir group than in the oseltamivir group in both subgroups (both *p* < 0.05), and there was no subgroup difference (*p* = 0.230, Figure 3C). Regarding influenza virus A subtypes H1N1pdm09 and H3N2A, subgroup analyses on the duration of fever, based on 5 and 3 studies, respectively, reported this endpoint. Heterogeneity was not detected in these two subgroups (both *p* > 0.05). The duration of fever was shorter in the baloxavir group than in the oseltamivir group in the influenza virus A subtype H1N1pdm09 subgroup (*p* < 0.001) but not in the influenza virus A subtype H3N2A subgroup (*p* = 0.430). There was no difference between these two subgroups (*p* = 0.140, Figure 3D).

### 3.5 Comparison of the safety of baloxavir and oseltamivir

**Figure 4:**
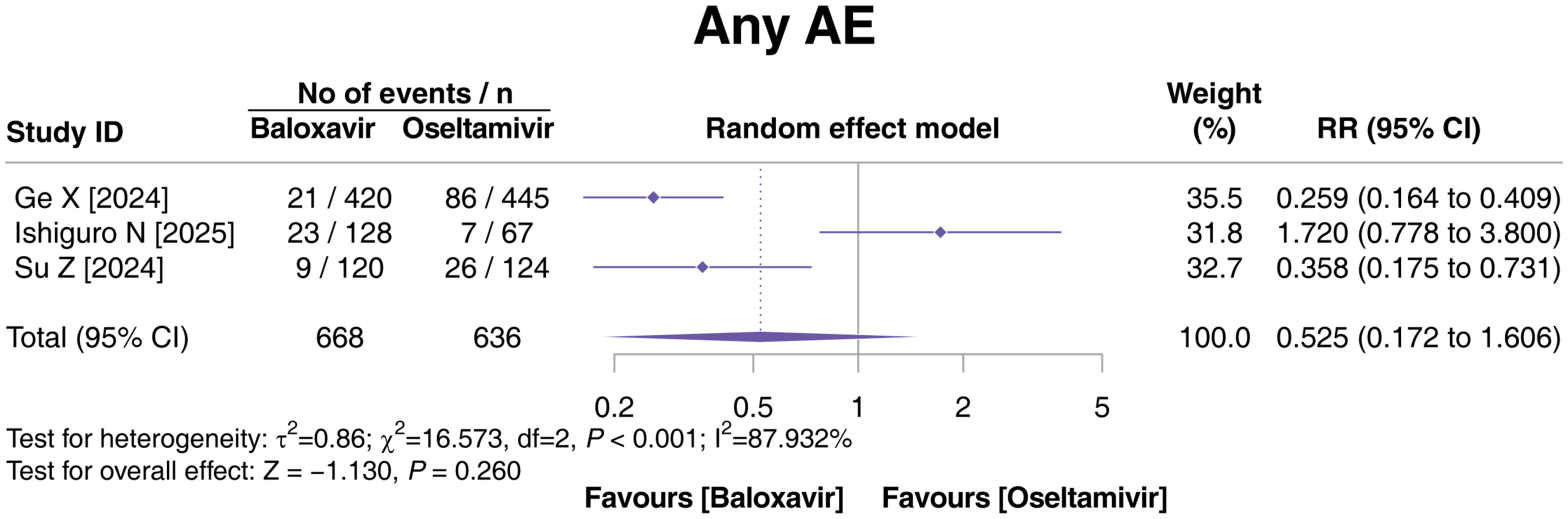
Comparison of any AEs between baloxavir and oseltamivir in pediatric patients with influenza.

Three studies reported AEs, and there was heterogeneity among these three studies (*p* < 0.001, *I*^2^ = 87.932%). The incidence of any AE was not different between the baloxavir and oseltamivir groups (*p* = 0.260, Figure 4).

### 3.6 Quality assessment and publication bias

The quality assessment was conducted on the RCT studies using the Cochrane ROB 2.0 tool and on the non-RCT studies using the Newcastle–Ottawa scale. The Cochrane ROB tool 2.0 assessed five domains: domain 1, bias arising from the randomization process; domain 2, bias due to deviations from intended interventions; domain 3, bias due to missing outcome data; domain 4, bias in measurement of the outcome; and domain 5, bias in selection of the reported result. The findings indicated that these two RCTs presented low risk across all domains (Table 2); moreover, the Newcastle–Ottawa scale was applied to evaluate the quality of non-RCT studies, including the dimensions of selection, comparability, and outcome. A study can be awarded a maximum of one star for each numbered item within the selection and exposure categories. A maximum of two stars can be given for “comparability.” Therefore, the highest number of stars for each study was 9, with more stars indicating higher quality. These non-RCT studies were awarded 8–9 stars for the items of selection, comparability, and outcome (Table 3). These findings indicated the high quality of the RCT and non-RCT studies.

**Table 2:** Quality assessment was performed via the Cochrane ROB tool 2.0.

No. of study	Domain 1	Domain 2	Domain 3	Domain 4	Domain 5
S1 (Baker et al., 2020)	Low risk	Low risk	Low risk	Low risk	Low risk
S4 (Ishiguro et al., 2025)	Low risk	Low risk	Low risk	Low risk	Low risk

Domain 1: bias arising from the randomization process.

Domain 2: bias due to deviations from intended interventions.

Domain 3: bias due to missing outcome data.

Domain 4: bias in the measurement of the outcome.

Domain 5: bias in the selection of the reported result.

**Table 3:** Quality assessment via the Newcastle–Ottawa scale.

No. of study	Selection				Comparability	Outcome		
	1	2	3	4	1	1	2	3
S2 (Chong et al., 2021)	★	★	★	★	★	★	★	★
S3 (Ge et al., 2024)	★	★	★	★	★★	★	★	★
S5 (Kakuya et al., 2022)	★	★	★	★	★★	★	★	★
S6 (Nezu et al., 2023)	★	★	★	★	★	★	★	★
S7 (Saito et al., 2020)	★	★	★	★	★	★	★	★
S8 (Sun et al., 2024)	★	★	★	★	★★	★	★	★
S9 (Wagatsuma et al., 2022)	★	★	★	★	★	★	★	★
S10 (Su et al., 2024)	★	★	★	★	★	★	★	★

A study can be awarded a maximum of one star for each numbered item within the “Selection” and “Exposure” categories. A maximum of two stars can be given for “Comparability.” Available from: http://www.ohri.ca/programs/clinical_epidemiology/oxford.asp.

**Figure 5:**
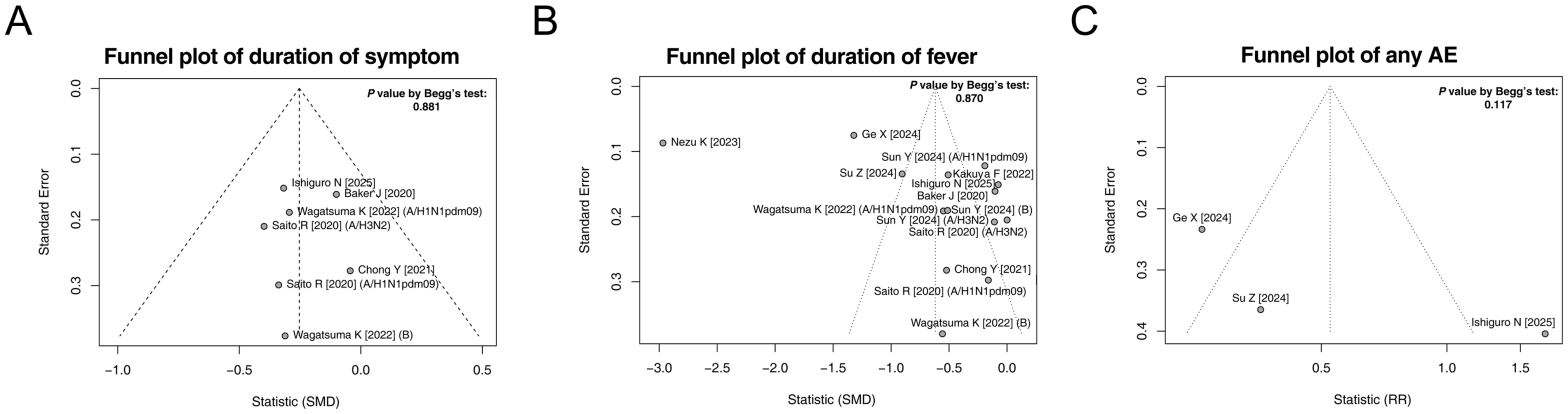
Funnel plots and publication bias. Funnel plots and publication bias of the duration of symptoms **(A)**, duration of fever **(B)**, and any AE **(C)**.

Begg’s test and funnel plots were used to assess publication bias in terms of the duration of symptoms, duration of fever, and incidence of any AE. These results indicated that there was no publication bias in these three outcomes (all *p* > 0.05, Figures 5A–C).

## 4 Discussion

This study revealed several interesting findings: (1) Baloxavir resulted in a shorter duration of symptoms and fever than did oseltamivir, but heterogeneity existed among the studies regarding the duration of fever. (2) Heterogeneity regarding the duration of fever may exist in influenza A virus subtypes H1N1pdm09 and H3N2A on the basis of subgroup analysis. (3) The safety profiles of baloxavir and oseltamivir were similar.

Compared with adults, pediatric patients with influenza have a higher susceptibility, higher incidence, and longer viral shedding durations, which leads to a worse prognosis. Therefore, the treatment of pediatric patients with influenza has received increasing attention from clinicians (Vasilakopoulos and Kainth, 2025; Goldman, 2021). Neuraminidase inhibitors, such as oseltamivir, are widely applied in the treatment of pediatric patients with influenza. However, oseltamivir needs to be used twice daily for five consecutive days, which is relatively inconvenient for pediatric patients with influenza. As an anti-influenza drug, baloxavir requires only a single dose throughout the treatment course, thereby improving treatment compliance (Ishiguro et al., 2025; Palmu et al., 2025). Moreover, several recent studies have shown that baloxavir is superior to oseltamivir in terms of efficacy (Ishiguro et al., 2025; Nezu et al., 2023). Consistent with these findings, this meta-analysis revealed that baloxavir exhibited a shorter duration of symptoms and fever than did oseltamivir. These findings could be explained as follows: (1) The antiviral mechanism of baloxavir is to inhibit the synthesis of viral mRNA, while oseltamivir inhibits only the release of the virus but does not reduce the virus load (Abed and Boivin, 2017). (2) Drug resistance-related genes were more prevalent for oseltamivir, whereas this resistance seemed to be less prevalent for baloxavir (Raza and Ashraf, 2024; Xu et al., 2024). (3) Although the bioavailability of oseltamivir is approximately 80%, which is greater than that of baloxavir (approximately 50%), the half-life of baloxavir is as high as 80–100 h, which is greater than that of oseltamivir (approximately 6–10 h), which might contribute to the better efficacy of baloxavir than that of oseltamivir (Heo, 2018). These findings support its superior efficacy to that of oseltamivir, which could be an alternative for pediatric patients with influenza.

The subgroup analysis in this study indicated that the heterogeneity in the duration of fever might be derived from influenza virus A subtypes H1N1pdm09 and H3N2A; moreover, the difference in efficacy between baloxavir and oseltamivir was found only in influenza virus A subtype H1N1pdm09, but not in subtype H3N2A. These findings might be due to (1) their distinct mechanisms of action: baloxavir targets the polymerase acidic protein endonuclease subunit, which is highly conserved in H1N1pdm09, whereas oseltamivir-mediated neuraminidase inhibition results in more resistance mutations (such as H275Y) in this clade (Li et al., 2013; Grund et al., 2015). (2) Second, this phenomenon might be because H3N2 maintains greater neuraminidase stability for oseltamivir targeting, leading to the development of polymerase acidic protein mutations (e.g., I38T) that reduce the effectiveness of baloxavir, narrowing the efficacy gap between the two drugs for H3N2 infections (Takashita et al., 2019). The findings from this study were also consistent with those of previous studies (Wagatsuma et al., 2022; Kakuya et al., 2022), suggesting that the benefit of baloxavir may vary among different virus subtypes. Therefore, the choice of specific anti-influenza drugs could be considered after detailed verification of the virus subtype.

Apart from single-drug administration, the double-drug regimen exhibits synergistic effects, indicating better efficacy than the single-drug regimen. For example, the double-drug regimen with oseltamivir and itraconazole exhibits stronger antiviral activity than monotherapy with oseltamivir (Schloer et al., 2020). Similarly, the double-drug regimen with the MEK inhibitor ATR-002 and baloxavir also shows synergistic potency (Hamza et al., 2021). In addition, evidence from *in vitro*, *in vivo*, and clinical studies also supports the combination of oseltamivir and baloxavir for treating influenza (Guo et al., 2024; Koszalka et al., 2022; Kumar et al., 2022). In addition, the ongoing COMBO 1 study (NCT04327791) also suggests the potential of combining baloxavir and oseltamivir for hospitalized patients with influenza. However, to support the administration of baloxavir and oseltamivir in clinical practice, more evidence is needed.

Safety is an ultimate concern for pediatric patients with influenza. Recent studies have indicated that the incidence of AEs is greater for baloxavir than for oseltamivir (Ge et al., 2024; Su et al., 2024), whereas another study revealed that there is no difference in the incidence of AEs between these two drugs (Ishiguro et al., 2025). In this meta-analysis, the incidence of AEs was similar between baloxavir and oseltamivir. The difference in these findings between this meta-analysis and the previous studies was hypothesized to be that (1) the sample size was still small in these studies (Ge et al., 2024; Ishiguro et al., 2025; Su et al., 2024); therefore, it was difficult to draw a solid conclusion on the safety comparison between baloxavir and oseltamivir. (2) Although there was no publication bias according to the funnel plot and Begg’s test, the different study designs might still cause potential bias in the conclusions. In detail, these studies, which indicated that baloxavir was preferable to oseltamivir in terms of safety profiles, were retrospective. This study design might lead to incomplete data and selection bias (Ge et al., 2024; Su et al., 2024). This study revealed that baloxavir has a similar safety profile to that of oseltamivir, and an RCT provides additional evidence (Ishiguro et al., 2025). Therefore, more studies are needed to compare the safety profiles of baloxavir and oseltamivir to determine their safety outcomes.

Previously, a meta-analysis carried out by Zhu et al. (2025) revealed several findings similar to those of our study, but some differences in study inclusion, default outcome, final inclusion time of the literature, covered literature, and statistical plan between our study and the previous review by Zhu et al. (2025) were found. In detail, (1) in terms of study inclusion, in the study by Zhu et al. (2025), Hayden et al. (2018), and Ison et al. (2020) were included, while these two studies included both adults and adolescents. The inclusion criterion for patients in the former was patients aged ≥ 12 to ≤ 64 years, whereas for the latter, it was over 12 years (Hayden et al., 2018; Ison et al., 2020). When the full texts and attachments of the data were screened, no separate subgroup data for the group under 18 years of age were retrieved; therefore, these two papers were excluded from our study. (2) In terms of the default outcome, the main default outcome was the duration of symptoms and fever in our study. However, the Sato et al. (2021) study did not report the duration of symptoms or fever, which was excluded from our study, but was included in the study by Zhu et al. (2025). (3) In terms of the final inclusion time of the literature, it was June 2025 in our study, but 25th December 2024, in the study by Zhu et al. (2025). (4) A total of 8 studies were included in the Zhu et al. (2025) study, 3 of which were excluded for the abovementioned reasons. The remaining five studies were included in our study. In addition to these five studies, five more studies were included in the current study. (5) In terms of the statistical plan, in addition to the main finding (comparison of the efficacy of baloxavir and oseltamivir), subgroup analysis was carried out in our study to explore the factors associated with the efficacy of baloxavir.

Several limitations in this study are unavoidable. (1) Most studies were performed in Japan or China; therefore, regional bias might exist. However, further global studies are needed. (2) Eight studies were non-RCT studies, and the inherent limitations of non-RCT studies still exist, such as selection bias, cofounders, and missing data. (3) As the data were derived from only 10 studies in this meta-analysis, the number of patients was small, limiting the statistical power to draw solid conclusions, such as the safety profile. (4) Patient comorbidities, such as coinfections, can significantly influence symptom severity and hospitalization duration; however, due to the lack of data, it was difficult to perform subgroup analysis. (5) Several important clinical parameters, such as length of hospital stay, oxygen saturation, intensive care unit (ICU) admission, and vaccine use, were not reported in the included papers, which might impact the efficacy of baloxavir. Therefore, further studies are needed to verify this finding. (6) Even though this study revealed that the efficacy of baloxavir against H3N2 was reduced, owing to the limited data and potential biases, the reliability of this information was limited. However, further studies are needed to verify this conclusion. (7) Some published studies, including Hayden et al. (2018), Ison et al. (2020), and Sato et al. (2021), were not included in this meta-analysis. Moreover, some ongoing and unpublished studies (such as NCT06762587) do not report their results. These findings might further impact the conclusions of the current study. Further meta-analysis could be conducted, incorporating more published findings, to draw a more solid conclusion. (8) The time from symptom onset to drug administration was hard to compare between baloxavir and oseltamivir due to the lack of available data in this study, which was hypothesized to have a major impact on the clinical outcome. Further study could be conducted to assess the impact of the time from symptom onset to drug administration on the duration of symptoms.

In conclusion, baloxavir is associated with a shorter duration of symptoms and a shorter duration of fever compared to oseltamivir, which may be attributed to their different bioavailability and half-lives. Meanwhile, the safety profiles are similar between these two drugs in pediatric patients with influenza. Further studies are still needed to verify these findings.

## Data Availability

The original contributions presented in the study are included in the article/supplementary material, further inquiries can be directed to the corresponding author.
