# Supplementary material for: Comparison of the efficacy and safety of baloxavir versus those of oseltamivir in pediatric patients with influenza: a meta-analysis
Source: Front Microbiol. 2025 Oct 28;16:1672925. doi: 10.3389/fmicb.2025.1672925 (PMC12602513; doi:10.3389/fmicb.2025.1672925)
Supplement: Supplementary file 1 [file Table_1.DOCX]

***Supplementary Materials***

**Supplementary Table 1.** Additional information on included studies.

| No. of study | Age (years) | | Time from symptom onset to drug administration | | Duration of medication use (days) | |
| --- | --- | --- | --- | --- | --- | --- |
|  | Baloxavir | Oseltamivir | Baloxavir | Oseltamivir | Baloxavir | Oseltamivir |
| S1 [17] | 6.1±2.9 | 6.0±3.2 | NA | NA | 1 | 5 |
| S2 [23] | 8.2±1.7 | 4.6±2.7 | Mean: 19.6 hours | Mean: 22.3 hours | 1 | 5 |
| S3 [16] | 7.3 (0.5-17.2) # | 11.5 (2.8-17.3) # | Median: 24.0 hours | Median: 27.0 hours | 1 | 5 |
| S4 [24] | 9.0±1.6 | 9.0±1.6 | ≤48 hours | ≤48 hours | 1 | 5 |
| S5 [25] | 10.0 (8.0-12.7) | 5.7 (4.2-7.5) | Median: 18.2 hours | Median: 18.2 hours | 1 | 5 |
| S6 [26] | 3.0±0.1* | 2.5±0.1* | <24 hours: 474 cases;  24-48 hours: 81 cases | <24 hours: 474;  24-48 hours: 82 | 1 | 5 |
| S7 [18] | 10.6±2.7 | 6.9±2.9 | Median: A(H1N1)pdm09: 14.5 hours;  A(H3N2): 15.0 hours | Median: A(H1N1)pdm09: 17.0 hours;  A(H3N2): 13.0 hours | 1 | 5 |
| S8 [19] | A(H1N1)pdm09:10.0 (8.0-12.0);  A(H3N2): 10.0 (8.0-12.0);  B: 9.0 (7.0-11.3) | A(H1N1)pdm09: 5.0 (3.0-7.0);  A(H3N2): 5.0 (3.8-7.0);  B: 6.0 (5.0-7.0) | A(H1N1)pdm09: 84 cases ≤24 hours;  A(H3N2): 21 cases ≤24 hours;  B: 28 cases ≤24 hours | A(H1N1)pdm09: 208 cases ≤24 hours;  A(H3N2): 430 cases ≤24 hours;  B: 164 cases ≤24 hours | 1 | 5 |
| S9 [20] | 9.8 (7.2-12.2) | 5.8 (4.2-8.3) | Mean: A(H1N1)pdm09: 12.4 hours;  B: 15.5 hours | Mean: A(H1N1)pdm09: 14.0 hours;  B: 19.7 hours | 1 | 5 |
| S10 [27] | 8.1 (6.5-10.8) | 7.9 (6.5-10.3) | NA | NA | 1 | 5 |

NA, not available. Age was described by mean±standard deviation or median (interquartile range), unless otherwise specified: the ‘#’ represented the median (range) and the ‘*’ represented the mean±standard error.
